# Rare subtype(s) and frequency of multi-viral subtype high-risk human papilloma virus infection in high-grade squamous intraepithelial lesion and squamous cell carcinoma in a cervical carcinoma prevalent developing country: a multiplex real-time polymerase chain reaction-based pilot study

**DOI:** 10.1186/s13000-016-0542-y

**Published:** 2016-09-22

**Authors:** Shabnam Akhter, Pradip Manna, Mohammed Kamal, C. James Sung, W. Dwayne Lawrence, M. Ruhul Quddus

**Affiliations:** 1 Department of Pathology, Bangabandhu Sheikh Mujib Medical University, Dhaka, Bangladesh; 2 Physicians Reference Laboratory, Overland Park, KS USA; 3 Department of Pathology, Women & Infants Hospital & Alpert Medical School of Brown University, 101 Dudley Street, Providence, RI 02905 USA

**Keywords:** High-risk HPV, Rare subtypes, Multi-subtype infection, Cervical carcinoma, Cervical dysplasia

## Abstract

**Background:**

Bangladesh, with a population of 160 million and nearly half being women, has the 4th highest rate of cervical carcinoma deaths in the world. It is projected that ∼500,000 of these women would die of this entirely preventable cancer by 2030. HPV vaccination is not widely offered in Bangladesh. This pilot study is designed to find out the prevalence of rare and multi-viral high-risk HPV (hrHPV) subtype(s) infection which may help strategize a large scale vaccination program in tackling cervical carcinoma in the country.

**Methods:**

Forty cases of cervical High-Grade squamous intraepithelial lesion (HSIL) and Squamous cell carcinoma (SqCa) were collected. DNA was extracted from tissue representing HSIL and SqCa and multiplex PCR was run to identify all 15 hrHPV subtypes along with known positive controls.

**Results:**

Of the total, 27 cases were biopsies/cones and 13 were hysterectomies including 5 HSIL and 35 SqCa. Infection caused by rare subtypes, hrHPV 45 and 52, were found in only two cases. Multi-subtype infection, detected in 28 % cases, was limited to HPV16/18 in all cases but one; one case showed hrHPV16/52 combination.

**Conclusion:**

A remarkable homogeneity of hrHPV 16 infection is noted in women with HSIL & SqCa in this country in these limited samples. This finding is in sharp contrast to the reports from western countries of frequent multi-viral and rare subtype hrHPV infection. This pilot study suggests that a vaccination program may be highly effective in controlling cervical cancer there. A larger study, however, is required to ratify the findings.

## Background

Cervical Cancer Global Crisis Card reports that Bangladesh ranks 4th in cervical carcinoma deaths in the world after India, China and Brazil (WHO). Incidence of cervical cancer is 19.2 per 100,000 women in Bangladesh [1]. The country is densely populated with 54.38 million women aged 15 years or older who are at risk of developing cervical cancer [2]. By 2030 an estimated 500,000 women in the middle and low income countries will die of this disease (WHO). According to a published report an estimated 6582 women per 100,000 die of cervical cancer annually (crude mortality rate of 8.7 %) [3, 4].

Human papillomavirus (HPV) infection causes cervical cancer and its precursor lesions in majority, if not all, cases [5]. Persistent infection with one or more types of high-risk HPV (hrHPV) is considered crucial in disease progression from cervical intraepithelial neoplasia (CIN) to invasive carcinoma [6]. More than 170 types of HPV have been identified until now [7], of which 40 distinct HPV types are known to date to infect the genital tract [8]. A meta-analysis of epidemiological studies designated 15 HPV types as high-risk types (16, 18, 31, 33, 35, 39, 45, 51, 52, 56, 58, 59, 68, 73, and 82) because of their significant association with progression to invasive cervical carcinoma (ICC), and additional three (26, 53, and 66) as *probable* high-risk types [9].

International Agency for Research on Cancer (IARC) reported HPV 16 to be the most common type worldwide followed by HPV 18, and together accounting for >70 % of ICC cases [10]. Epidemiological studies have shown that type-specific prevalence of HPV in cervical cancers differs according to geographical regions [11]. The authors reported that HPV 16 to be the predominant subtype ranging from 45.9 % in Asia to 62.6 % in Europe. The second most frequent subtype, HPV 18, was reported in approximately 10–14 % [10]. World-wide HPV 45, 31 and 33 were the next most frequent types, except in Asia where HPV 58 and 52 were found to be more frequent [10]. In another study, Bao et al. [12] reported ten most common HPV subtypes detected in invasive cervical carcinoma in Asia, e.g., HPV16, 18, 58, 33, 52, 45, 31, 35, 59, and 51.

Only a handful of studies are available in the literature on the prevalence of hrHPV in Bangladesh. These studies were carried out in selected patient population, such as, in female sex workers [13] and women with abnormal cervical cytology in a tertiary care hospital [14]. Recently Nahar et al. [15] reported a study on women recruited from an urban center and a rural center. All, except one, of these studies isolated HPV from cytologic preparation without any histologic correlation. Banik et. al [14] attempted to isolate HPV 16 and 18 from dysplastic tissue.

Multi-viral hrHPV infection has been reported to be associated with cervical dysplasia [16] and carcinoma [17]. It has been recently reported that as many as five different sub-types of hrHPV are being present in high grade dysplasia at a single anatomic location [18]. The frequency of multi-viral infection in dysplasia/neoplasia is not known in Bangladesh.

The current study attempts to identify the prevalence of all 15 hrHPV subtypes in HSILs and SqCa, presence of rare subtypes, and determine the frequency of multi-viral subtype infection from lesional tissue. This information would fill-up the void that exists in epidemiological data now present on HPV infection in this country. This information may also prove beneficial for the mass scale HPV vaccination program in controlling HPV infection and eventually saving numerous young lives in this country.

## Methods

This is a retrospective collaborative pilot study. The collaborating departments are the Departments of Pathology, Women & Infants Hospital/Alpert Medical School of Brown University, Providence, RI, USA; BSM Medical University (BSMMU), Dhaka, Bangladesh; and Physicians Reference Laboratory, Overland Park, KS, USA.

After obtaining approval from the Institutional review board of BSMMU, Dhaka, Bangladesh and Institutional Review Board of Women & Infants Hospital, 40 cases comprising of 35 SqCa and 5 cervical HSIL (CIN 2/3) diagnosed during August 2013 – January 2014 at the Department of Pathology of BSMMU, were selected for the study. Paraffin embedded tissue blocks were retrieved from the archive of Department of Pathology, BSMMU and sent to the Women & Infants Hospital, Providence, RI, USA without any patient unique identifiers. At Women & Infants hospital, Hematoxylin and eosin stained sections from each case were reviewed by one pathologist (MRQ) to confirm the diagnosis and affirm the presence of lesional areas for DNA extraction.

Five to 10, 5 μm tissue sections were obtained to extract DNA from the selected areas of formalin-fixed paraffin embedded (FFPE) tissue samples by using QIAmp DNA FFPE Kit. HPV testing was done using the proprietary COMPLeTe Care HPV test (Physicians Reference Laboratory, Overland park, KS), a multiplex real-time PCR test that simultaneously detected, typed and quantified all 15 high-risk HPV (hrHPV) subtypes. The tested subtypes were 16, 18, 31, 33, 35, 39, 45, 51, 52, 56, 58, 59, 68, 73, and 82, as reported by a consensus from several epidemiologic studies [9]. The method has been described by these authors in a previously published paper [17]. The target of COMPLeTe Care HPV test was *E7*, an oncogene in the HPV genome, which usually gets integrated into the host chromosome and plays a critical role in cervical carcinogenesis. The oncogenes *E6* and *E7* are stable unlike capsid gene *L1*, which is reported to be lost during integration.

Both absolute viral load and cell numbers were calculated from the quantitative standards included in each run with the patient samples. The viral loads and ß-globin amounts were determined on the basis of the crossing point (Cp) or crossing threshold (Ct) values above their corresponding baselines in relation to the quantitative standards [17]. Viral loads were normalized to 1000 cells. The quantification range of the assay is 10^1 to 10^6 copies/reaction and the qualitative lower limit of detection is <10 copies/reaction. Copies <10 are qualified simply as “positive”.

## Results

A total of 40 cases were studied, including 35 SqCa and 5 HSIL. The age distribution is shown in Table 1. The age range of the patients is 25–70 years, with a mean of 48.38 years and a median of 50 years (SD = 10.175). Of the total 40 specimens, 27 (67.5 %) were cervical cones/LEEPs or biopsies, and 13 (32.5 %) hysterectomies.

**Table 1:** Clinicopathologic data of the patients

Histological diagnosis	Grade of the lesion	*N* (%)	Stratified by age groups		
			<30 years	30–44 years	≥45 years
HSIL (*N* = 5)	CIN 2	1 (2.5 %)	-	1	-
	CIN 3	4 (10 %)	-	-	4
SqCa (*N* = 35)	Grade I	2 (5 %)	-	1	1
	Grade II	18 (45 %)	1	3	14
	Grade III	15 (37.5 %)	-	7	8

Note: *HSIL* High grade squamous intraepithelial lesion, *SqCa* Squamous cell carcinoma

All but one case (*N* = 39) in this series were successfully amplified by multiplex PCR indicating the hrHPV identification rate of 97.5 % in SqCa and HSIL specimens in this study. HPV 16 was detected in 38 of 39 cases (97.4 %), either alone or in combination with HPV 18 (25.6 %). HPV 18 has been identified only in combination with HPV 16. Rare subtypes, HPV 52 and HPV 45, were identified only in two cases. HPV 52 was identified in combination with HPV 16 and HPV 52 (2.5 %). The remaining case had a single infection by HPV 45.

Of the 35 cases of SqCa, DNA was amplified in all but one case. Out of the HPV positive 34 cases, 33 (97 %) had infection with HPV 16, either alone (*N* = 23) or in combination with HPV 18 (*N* = 9), or in combination with HPV 52 (*N* = 1). The single remaining case (2.9 %) showed amplification of only HPV 45. Thus, of the total of 34 SqCa, HPV 18 was present in 9 (26.5 %) patients and HPV 52 in only one patient (2.9 %), both as multiple subtype infections.

All the five cases of HSIL had infection with HPV 16 (100 %), and one of them also had co-infection with HPV 18 (20 %).

Multi-subtype infection, mainly of HPV 16/18, was present in 11 out of 39 (28 %) cases; ten of these were SqCa and one was a case of HSIL. The normalized viral load of each HPV subtype per 1000 cells is shown in Tables 2 and 3 shows the comparison of normalized viral loads between HSIL and SqCa.

**Table 2:** Normalized hrHPV viral loads

Subtypes	*N*	Viruses per 1000 cells
		Range
HPV 16	37	Positive*–440,991
HPV 18	10	Positive*–751
HPV45	1	2100
HPV 52	1	22

Note: *Copies <10 are qualified as “positive”

**Table 3:** Comparison of normalized viral loads in CIN vs. invasive carcinoma

Diagnosis	hrHPV Subtypes	*N*	HPV Viruses per 1000 cells
			Range
H*G*SIL (*N* = 5)	HPV 16	5	2018–1,529,730
	HPV 18	1	-
Inv SqCa (*N* = 35)	HPV 16	33	Positive*–4,409,910
	HPV 18	7	Positive*–751
	HPV 45	1	-
	HPV 52	1	-

Note: *Copies <10 are qualified as “positive”

## Discussion

Cervical carcinoma induced mortality and morbidity is high and it is the second highest cause of cancer-related deaths in women in Bangladesh [3]. HPV vaccination has recently been introduced in the country and sparingly offered to young women. The bivalent vaccine offers protection against hrHPV 16 and 18. It also offers some cross protection against HPV 45, 31, and 33 [19, 20]. The quadrivalent vaccine offers protection against HPV 6, 11, 16, and 18 and some cross protection against HPV 31, 33, 35, 39, 52, 58, and 59 [19–21].

As expected, hrHPV was detected in 97.5 % cases of SqCa and HSIL examined. One test (2.5 %) failed which could very well be attributed to any technical glitch. HPV 16 was detected in overwhelming majority of cases (38 of 39; 97.4 %), either alone or in combination with HPV 18 and HPV 52. Interestingly HPV 18 was identified only in combination with HPV 16 in 10 cases (25.6 %). Only in two cases, rare subtypes, e.g., HPV 52 and HPV 45 were identified. HPV 52 was identified in combination with HPV 16. The remaining one case had a single stand-alone infection by HPV 45. Although the rare subtypes HPV are detected in fewer numbers of cases now, they may potentially play an important role in future in post-vaccination era. Of note that some cross protection may be expected against these two rare subtypes of HPV by the quadrivalent and bivalent HPV vaccines available in the country. Recently Nahar and her colleagues reported HPV 66 in the country [15]. Of note, no vaccine available now or in developmental phase (the 9-valent vaccine) offers any protection against hrHPV 66.

The current study has found that the rare hrHPV subtypes are highly uncommon in this cervical carcinoma prevalent country. A remarkable homogeneity of HPV 16 infection (97.4 %) in Bangladeshi women with cervical SqCa and HSIL has been documented in this pilot study. This is significantly different from the epidemiologic data reported from the western countries where rare HPV subtypes are more frequently encountered [18]. A larger study is needed to ratify the findings of this pilot study.

Multi-subtype HPV infection was detected in 28 % cases in this study. Fortunately, this was noted only as a combination of HPV 16/18 and a single case of HPV16/52. Multiple HPV subtypes infection consisting of rare subtypes of HPV are more frequently reported in studies from Western countries [18]. One can speculate that the social and cultural practices may be the cause for this apparent difference of findings.

Viral load of hrHPV is found to have significant association with the development of HSIL and the risk is said to be enhanced with higher baseline HPV DNA [22]. Viral load may thus serve as an indicator of risk of disease progression, although lower viral load does not necessarily mean lack of progression [19] or viral clearance with no disease [23]. Individual immunity must play a role in the pathogenesis of the disease. As expected and shown in Table 3, the viral loads of HPV 16 in HSILs were much higher than that of SqCa cases. The HPV 16 viral copies were much higher in HSIL (5329–152,973) than those in SqCa (Positive–440,991), except in one case of SqCa which has the highest viral load of 440,991/1000 cells. This finding is similar to what is reported by Hui et al. [18]. The viral copies of the two rare hrHPV subtypes were much lower compared to HPV 16. Quddus et.al [17] previously reported that a small number of recurrent tumors had higher viral loads in their primary tumors.

Routine screening has successfully decreased hrHPV related female genital tract lesion in the Western world. But Bangladesh, being a developing nation, lacks comprehensive infrastructure for mass screening and early intervention of HPV infection. Therefore, mass immunization against the most prevalent hrHPV subtypes may be an effective way to address the issue. Fortunately even with its limited resources, Bangladesh has established an efficient infrastructure for grass-root level immunization program and has shown tremendous success in childhood vaccination program. Without enduring additional cost it appears possible that Bangladesh can bring down cervical cancer deaths using the existing infrastructure of childhood immunization program. Knowing specific hrHPV subtypes in dysplastic and neoplastic tissues would help the health care workers to fight against the current subtype(s) induced disease and also prepares for future should or when the rare subtype(s) emerge.

## Conclusions

A remarkable homogeneity of hrHPV 16 infection is noted in women with HSIL & SqCa in this country in this pilot study. This finding is in sharp contrast to the reports from western countries of frequent multi-viral and rare subtype hrHPV infection. This study suggests that a vaccination program would be highly effective in controlling cervical cancer should a larger study ratify the findings of this pilot study.
